# (*9R*)-9-Hydroxystearate-Functionalized Anticancer Ceramics Promote Loading of Silver Nanoparticles

**DOI:** 10.3390/nano8060390

**Published:** 2018-05-31

**Authors:** Elisa Boanini, Maria Cristina Cassani, Katia Rubini, Carla Boga, Adriana Bigi

**Affiliations:** 1 Department of Chemistry “Giacomo Ciamician”, University of Bologna, via Selmi, 2, 40126 Bologna, Italy; katia.rubini@unibo.it; 2 Department of Industrial Chemistry “Toso Montanari”, Via del Risorgimento, 4, 40136 Bologna, Italy; maria.cassani@unibo.it (M.C.C.); carla.boga@unibo.it (C.B.)

**Keywords:** hydroxyapatite, silver, nanoparticles, X-ray diffraction, transmission electron microscopy, ceramic, biomaterial

## Abstract

Functionalization of calcium phosphates for biomedical applications has been proposed as a strategy to enrich the good osteoinductive properties of these materials with specific therapeutic characteristics. Herein, we prepared and characterized hydroxyapatite nanocrystals functionalized with an anticancer agent, (*9R*)-9-hydroxystearate (HSA), and loaded with an antimicrobial agent, namely silver nanoparticles (AgNPs). Nanocrystals at two different contents of HSA, about 4 and 9 wt %, were prepared via direct synthesis in aqueous solution. Loading with the antibacterial agent was achieved through interaction with different volumes of AgNPs suspensions. The amount of loaded nanoparticles increases with the volume of the AgNPs suspension and with the hydroxystearate content of the nanocrystals, up to about 3.3 wt %. The structural, morphological, and hydrophobic properties of the composite materials depend on hydroxystearate content, whereas they are not affected by AgNPs loading. At variance, the values of zeta potential slightly increase with the content of AgNPs, which exhibit a sustained release in cell culture medium.

## 1. Introduction

Calcium phosphate (CaP)-based biomaterials are among the most utilized systems in biomedical applications aimed to solve problems related to musculoskeletal disorders. In particular, hydroxyapatite (HA) closely resembles the inorganic phase of bone and is the most employed CaP for hard tissue substitution/repair. HA displays an extremely good biocompatibility and bioactivity, and it can be utilized as delivery system of active agents aimed to promote specific biological functions [1]. To this purpose, HA can be functionalized with bioactive ions, molecules, growth factors, and drugs, adding specific characteristics to the osteogenic properties of the calcium phosphate [2,3,4,5,6,7]. We have previously synthesized HA at different contents of (*9R*)-9-hydroxystearate (HSA) [8]. HSA derives from 9-hydroxystearic acid, an endogenous long-chain monohydroxyl fatty acid, which is able to downregulate tumor cell proliferation [9]. In fact, it was shown to provoke apoptosis of the osteosarcoma cell line U2OS via a mitochondrial pathway [10]. Moreover, especially in the (*9R*) enantiomeric form, it exerts an antiproliferative action on the colon cancer cell line HT29 [11]. HSA maintains its antitumor activity also when incorporated into HA, in agreement with the cytostatic and cytotoxic effect of HSA functionalized HA nanocrystals on osteosarcoma cell line SaOS2, which is modulated by HSA content of the composite material [9].

Herein, we explored the possibility to add a further functionalization to hydroxystearate containing HA nanocrystals through loading of silver nanoparticles (AgNPs). AgNPs should imbue antimicrobial properties to the functionalized nanocrystals, in agreement with their strong efficacy as broad-spectrum antibacterial agents [12,13]. As a matter of fact, AgNPs are widely employed as antibacterial agents in the biomedical field, as well as in several other applications, including water disinfection and cosmetics [14]. Their main applications in the biomedical field include wound dressing, catheters, and cardiovascular implants [15]. More recently, AgNPs have been raising increasing interest also for orthopedic applications [4,16,17,18]. In this study we synthesized hydroxyapatite nanocrystals at different hydroxystearate and AgNPs contents. To this aim, we loaded different amounts of AgNPs stabilized with low molecular weight polyethylenimine (PEI) on hydroxyapatite synthesized at different hydroxystearate contents. The results indicate that hydroxystearate functionalization influences AgNPs loading, which reaches contents up to about 3.3 wt % in the samples at the highest hydroxystearate content.

## 2. Materials and Methods

### 2.1. Synthesis of Composite Materials

Hydroxyapatite was synthesized by a co-precipitation method, using CO_2_-free distilled water and under N_2_ atmosphere in order to avoid the formation of carbonated apatite. Fifty milliliters of Ca(NO_3_)_2_·4H_2_O solution (1.08 M), pH 10 adjusted with ammonia, was added drop-wise to 50 mL of (NH_4_)_2_HPO_4_ 0.65 M solution at 90 °C under stirring. The reaction solution was kept at 90 °C for 5 h under stirring, and then the precipitate was separated by centrifugation at 10,000 rpm for 10 min, rinsed twice with CO_2_-free distilled water, and dried at 37 °C.

Potassium (*9R*)-9-hydroxystearate was prepared by treatment of (*9R*)-9-hydroxystearic acid, obtained from *Dimorphotheca sinuata* L. seed oil [11], with an equimolar amount of KOH in methanol solution, as previously described [8]. Samples containing HSA were obtained following the above procedure but dissolving potassium (*9R*)-9-hydroxystearate into the (NH_4_)_2_HPO_4_ solution before starting dropping the Ca(NO_3_)_2_·4H_2_O solution. The concentrations of potassium (*9R*)-9-hydroxystearate used were 10 and 20 mM, calculated on final volume. The obtained powder samples were labelled as HSA10 and HSA20, respectively.

Silver nanoparticles (AgNPs) solution was prepared by heating 100 ml of AgNO_3_ solution (10 mM in ultra-pure MilliQ water) until 100 °C under stirring. When the solution is boiling, 0.70 mL 10% (*w*/*w*) of polyethyleneimine (PEI) (average M_w_ ~2000, 50 wt % in H_2_O, Sigma-Aldrich, St. Louis, MO, USA) solution was added quickly in one step. The solution was vigorously stirred for 4 min and then cooled at room temperature. Finally, the volume was taken back to 100 mL with ultra-pure water in order to compensate for evaporation.

In order to support AgNPs on apatitic materials, different volumes of AgNPs solution were added to 0.5 g of powder (HA, HSA10, and HSA20). Each support was submitted to incubation with 5, 20, and 50 mL of solution. The suspension was vigorously stirred for 1 h at room temperature. The product is then filtered and dried at 37 °C. Final materials were labeled using a combination indicating the kind of apatitic support and the volume of AgNPs solution (i.e., HA-Ag5 indicates the sample obtained after incubation of HA into 5 mL of AgNPs solution; HSA20-Ag50 indicates the sample obtained after incubation of HSA20 into 50 mL of AgNPs solution).

### 2.2. Characterization of Composite Materials

X-ray diffraction analysis was performed using a PANalytical X’Pert PRO powder diffractometer (Malvern PANalytical, Almelo, The Netherlands) equipped with a fast X’Celerator detector. CuKα radiation was used (40 mA, 40 kV). The patterns were recorded in the 10°–60° 2θ range with a step size of 0.1° and time/step of 100 s. In order to evaluate the coherence lengths of the crystals and to perform the full profile pattern refinement, further X-ray powder data were collected with a fixed counting time of 400 s for each 0.033/step, using silicon as internal standard. The Scherrer formula [19] was applied to calculate the coherence lengths of crystalline domains. The Rietveld routine of the HighScore Plus software package (Malvern PANalytical, Almelo, The Netherlands)) was used to process the data for evaluation of cell parameters.

HSA content was determined through thermogravimetric analysis using a Perkin–Elmer TGA-7. Heating was performed in a platinum crucible in air flow (20 cm^3^/min) at a rate of 10 °C/min up to 900 °C. The samples’ weights were in the range 5–10 mg.

The amount of silver present in the different samples was determined by atomic absorption spectroscopy (AAS, Thermo Scientific, Waltham, MA, USA) in air-acetylene flame (λ = 328.1 nm; spectral band-width = 0.5 nm). Ca. 8 mg of previously grinded solid samples were solubilized in 25 mL of a 0.5 M HNO_3_ aqueous solution. The calibration line was made with 5 calibration standards (2, 4, 6, 8, 10 ppm), prepared by dilution to 50 mL of a 100 ppm silver standard for AAS in 0.5 M HNO_3_ (Merck KGaA , Darmstadt, Germany).

A Philips CM100 transmission electron microscope (80 kV) was used for TEM investigations. Sample powders were suspended in ethanol and sonicated. A drop of sonicated suspension was transferred onto formvar films supported on conventional copper microgrids. The ImageJ^®^ picture analyzer software was used to estimate the mean particles dimensions. The reported results are the average values of measurements performed over at least 100 data points per sample.

Zeta potential was measured using Electrophoretic Light Scattering (ZetasizerNano; Malvern PANalytical, Malvern, UK). Five milligrams of powder sample were suspended in 50 mL of MilliQ water after sonication for 2 min. Each analysis was performed in triplicate.

Atomic force Microscopy (AFM), contact angle and silver release analyses were carried out on disk-shaped samples (Ø = 6.0 mm) in order to examine the materials mimicking the conditions of possible applications as biomaterials. Disk-shaped samples were prepared by pressing the powder (40 mg for each disk) into cylindrical molds by using a standard evacuable pellet die (Hellma, Müllheim, Germany).

Static contact angle measurements were performed by means of a Theta Lite optical tensiometer (Biolin Scientific, Gothenburg, Sweden) under ambient conditions by recording the side profiles of deionized water drops for image analysis. The shape of the drop was recorded in a time range of 0–30 s, with images collected every 0.033 s. At least five drops were observed for each sample.

AFM imaging was performed using a Veeco Nanoscope 3D instrument (Veeco, Plainview, NY, USA). The disk-shaped samples were analyzed in tapping mode using an E scanner (maximum scan size 15 μm) and phosphorus (n) doped silicon probes (spring constant 20–80 N/m; resonance frequency 250–290 kHz; nominal tip radius <10 nm). Roughness parameters, namely arithmetic mean roughness (Ra), root-square roughness (Rq), and the vertical distance between the highest and lowest points within the evaluation length (Rt), were recorded.

Release of silver from disk-shaped samples was measured in the medium used for cell culture differentiation, Dulbecco’s Modified Eagle Medium (DMEM, Sigma, Saint Louis, MO, USA) supplemented with antibiotics (100 U/mL penicillin, 100 μg/mL streptomycin). Ag content in the supernatant was analyzed at increasing times up to 14 days using flame atomic absorption spectroscopy (AAS, Thermo Scientific, Waltham, MA, USA), in air-acetylene flame (λ = 328.1 nm; spectral band-width = 0.5 nm). Results from this analysis represent the mean value of three different determinations.

## 3. Results and Discussion

**Figure 1:**
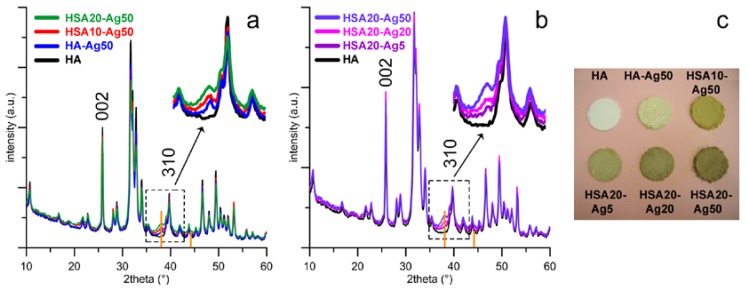
X-ray diffraction patterns of materials functionalized with silver nanoparticles (AgNPs): (**a**) different supports loaded in 50 mL of AgNPs suspension; (**b**) HSA20 loaded in different volumes of AgNPs; (**c**) photographs of samples. The samples are labelled HA-AgX and HSAY-AgX, where X indicates the volume (in mL) of the AgNPs suspension used for loading, and Y is the hydroxystearate concentration (in mM) in the synthesis solution. The positions of the 002 and 310 peaks of HA used for the evaluation of the crystallite size are indicated. The two orange bars indicate the positions of the two most intense reflections of silver metal (PDF 4-873).

The XRD patterns of the different supports functionalized with AgNPs show a number of reflections, which correspond to those characteristic of hydroxyapatite (PDF 9-432), together with the most intense reflection of Ag at about 38.1 ° of 2θ (PDF 4-873). The relative intensity of this peak increases on passing from HA-Ag50 to HSA10-Ag50, to HSA20-Ag50 (Figure 1a) and as the volume of the AgNPs colloidal suspension increases (Figure 1b), suggesting the presence of variable quantities of metallic silver associated to the different materials as a function of experimental procedures.

Functionalization does not affect significantly the dimensions of the unit cell of the apatitic structure, as shown by the values of the lattice parameters of the different samples reported in Table 1. The comparison between the XRD patterns reported in Figure 1a shows that the patterns of the samples functionalized with HSA display slightly broader diffraction reflections than those of HA-Ag50, in agreement with an overall reduction of the length of crystallite sizes (τ_hkl_) provoked by HSA functionalization [8]. The values of the mean crystallite sizes along the *c*-axis (τ_002_) and along a direction perpendicular to it (τ_310_), calculated using the Scherrer equation [19] and reported in Table 1, display an anisotropic reduction on increasing HSA content. The greater reduction along the direction perpendicular to the *c*-axis suggests that hydroxystearate is preferentially adsorbed on HA (hk0) faces, as previously reported for anionic polyelectrolytes and acidic amino acids [8,20,21].

**Figure 2:**
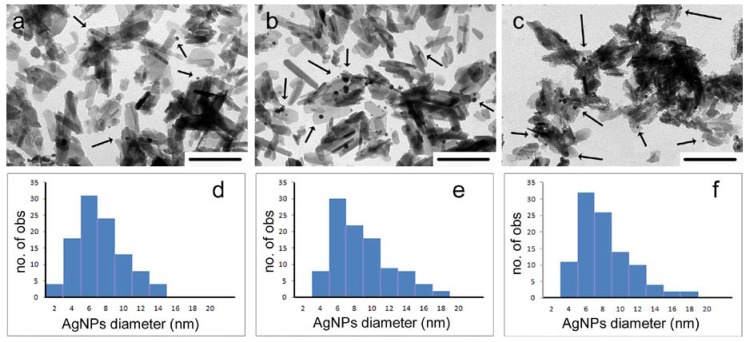
Transmission electron microscopy images of materials functionalized with AgNPs and related AgNPs size distribution diagrams: (**a**,**d**) HA-Ag50; (**b**,**e**) HSA10-Ag50 and (**c**,**f**) HSA20-Ag50. The arrows in micrographs indicate AgNPs. Bars = 200 nm.

Quantitative chemical analysis confirms that AgNP loading depends both on the different supports and on the amount of AgNPs suspension used for the loading procedure: as shown in Table 1, the maximum amount loaded on HA is about 1.2%, whereas it reaches values up to 3.3% on HSA containing supports. The maximum AgNPs loading achieved on HSA samples is smaller than that previously loaded on polyacrylate functionalized HA supports [22]. However, previous data demonstrated that contents greater than 3% provoke significant cytotoxicity, whereas lower values imbue the materials with a long standing antibacterial activity without causing cytotoxicity [22]. The different amounts of AgNPs loaded on the different substrates are confirmed by the different number of nanoparticles appreciable in the TEM images of the different samples. The images reported in Figure 2a–c allow to appreciate a significantly higher number of AgNPs on HSA containing samples than on HA nanocrystals. Furthermore, electron microscopy allows to verify that AgNPs are always associated to the surface of the apatitic crystals. In fact, free particles are never observed. The average diameter of the nanoparticles, about 7 nm, as well as their size distribution, does not vary significantly on the different supports (Figure 2d–f).

A qualitative evaluation of AgNPs presence is provided also by the color variation of the different supports which can be observed on increasing AgNPs loading (Figure 1c). The presence of silver provides the powders with a yellow-brownish color, which is more intense for HSA functionalized samples and becomes darker on increasing AgNPs content. The great AgNPs amounts loaded onto HSA-containing supports in comparison to HA support can be related to the more negatively charged surfaces of the functionalized crystals, which exert a greater attraction towards the PEI-stabilized AgNPs. Indeed, HSA10 and HSA20 exhibit more negative values of zeta potential than HA. Furthermore, adsorption of the positively charged nanoparticles (zeta potential of AgNPs = +37 mV [22,23]) provokes an increase of the zeta potential of all the samples, as shown in Table 1.

**Figure 3:**
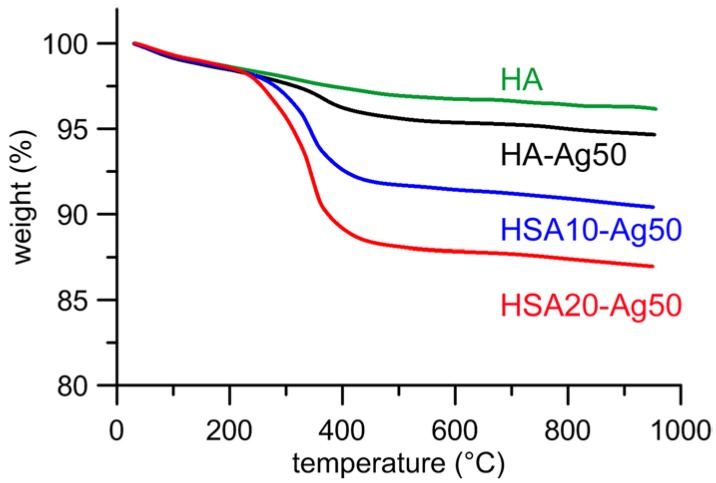
Thermogravimetric plots of as-prepared HA, HA-Ag50, HSA10-Ag50, and HSA20-Ag50, useful for quantification of HSA in the composite materials.

AgNPs adsorption does not provoke any significant variation of the content of HSA of the different samples, as determined through thermogravimetric analysis. Thermal decomposition of hydroxystearate occurs between 200 and 600 °C and the total weight loss calculated from the thermogravimetric plots of the different samples indicates that HSA content assumes mean values of about 4 and 9% respectively in HSA10 and HSA20 samples, independently from the presence of AgNPs. Typical thermogravimetric plots are reported in Figure 3 for samples loaded with Ag50. The slightly different weight losses (about 1 wt %) exhibited by HA-Ag50 and HA, which has been reported for comparison, are due to the adsorption of PEI from the AgNPs suspension onto HA-Ag50 nanocrystals.

**Figure 4:**
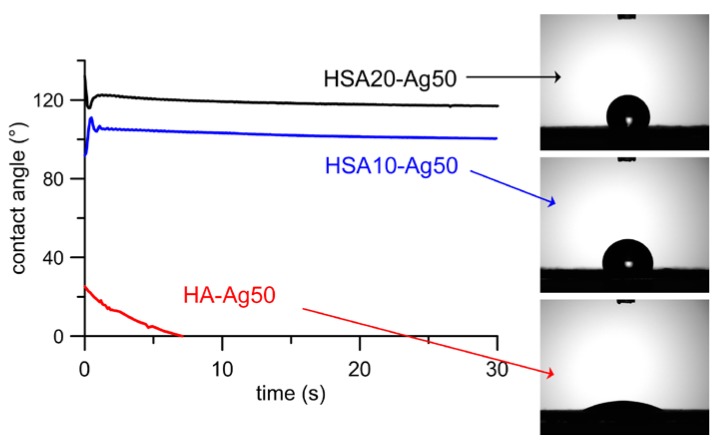
Plots of contact angle values as a function of time. The images of droplets measured after 1 second on different composite materials are shown on the right.

HSA hydrophobic tails on the nanocrystals surface are also responsible for the very great difference between the values of contact angle measured on HSA containing samples than on the HA ones (Table 1). The values are slightly higher for HSA20 than for HSA10 samples, in agreement with their different hydroxystearate content, and do not vary during the 30 s of acquisition. At variance, the water droplet is completely spread on HA samples within a few seconds (Figure 4). The data reported in Table 1 show that the hydrophobic/hydrophilic behavior of the samples was not significantly modified by the presence of AgNPs.

**Figure 5:**
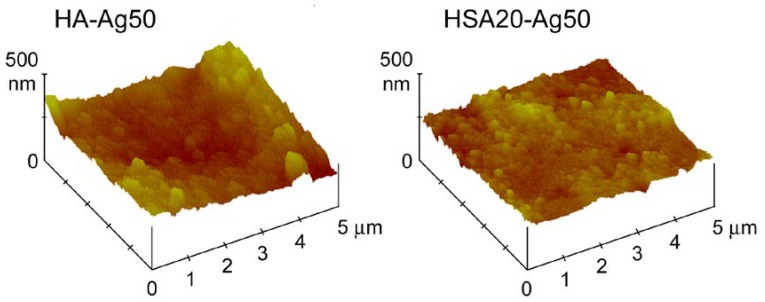
Atomic force microscopy images of the surfaces of disk-shaped samples obtained by pressing as-prepared HA-Ag50 and HSA20-Ag50 powders into cylindrical molds.

Contact angle measurements, as well as AFM investigation and release analysis, were performed on disk-shaped samples in order to mimic the conditions of possible applications as biomaterials. Indeed, the nanometric dimensions of the synthesized materials can cause severe cytotoxic effects and prevent their use as free powders [24]. The results of AFM investigation indicate a decrease of the roughness parameters, Ra, Rq and Rt, on passing from HA to HSA containing samples, most likely because of the smaller dimensions of these last nanocrystals, as it can be appreciated also in Figure 2. The average values do not vary significantly as a function of AgNPs content and are: Ra = 0.060 ± 0.004 µm, Rq = 0.075 ± 0.002 µm, Rt = 0.495 ± 0.015 µm for HA and Ra = 0.024 ± 0.002 µm, Rq = 0.030 ± 0.003 µm, Rt = 0.247 ± 0.012 µm for HAHSA20 samples, independently from their AgNPs content. The images of two typical samples are reported in Figure 5.

**Figure 6:**
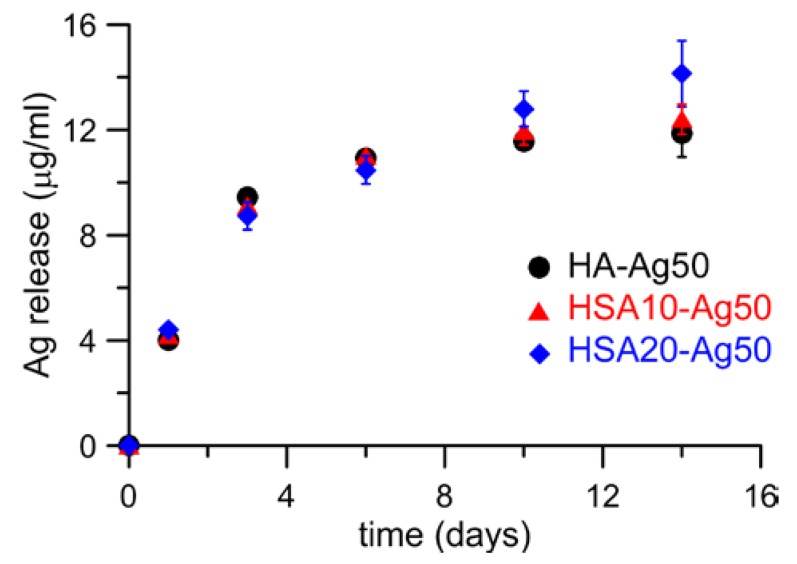
Silver cumulative release from HA-Ag50, HSA10-Ag50, and HSA20-Ag50 as a function of time.

Ag release from HA-Ag50, HSA10-Ag50, and HSA20-Ag50 was measured in Dulbecco’s Modified Eagle Medium up to 14 days. The results show that the cumulative release follows a similar trend for both type of supports, reaching values of about 12–14 μg in the first 2 weeks (Figure 6). This amount corresponds to about 2 wt % of the initial Ag content of HA-Ag50, whereas the percentage of release from HSA containing samples is just about 1 wt % of the initial content.

**Figure 7:**
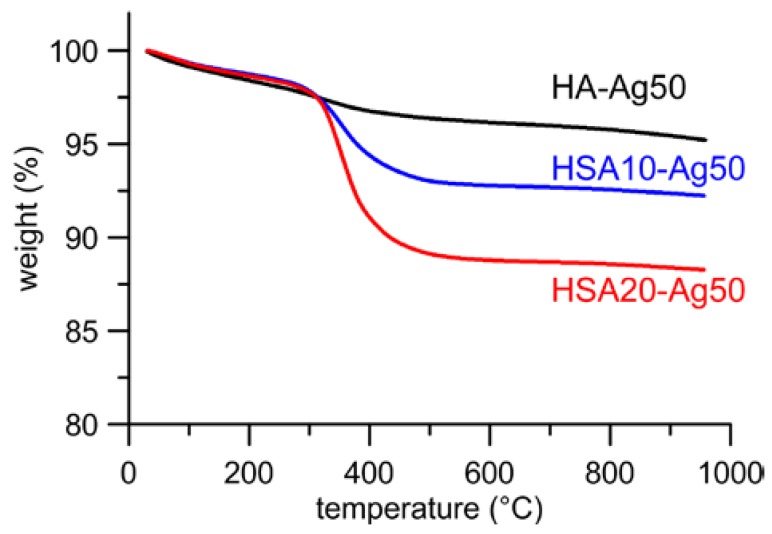
Thermogravimetric plots of HA-Ag50, HSA10-Ag50, and HSA20-Ag50 after 14 days soaking in DMEM.

At variance, the comparison of the thermogravimetric curves of the different samples before and after 14 days soaking in DMEM does not reveal significant difference in the total weight losses, in agreement with no significant hydroxystearate release from the samples (Figure 7). The results of Ag and hydroxystearate release are supported by the results of AFM analysis, which indicate that the morphology and the roughness parameters of the samples are not significantly affected by soaking in DMEM.

## 4. Conclusions

The results of this study indicate that functionalization of hydroxyapatite nanocrystals with hydroxystearate enhances AgNPs loading, most likely thanks to the interaction between the positive charges provided by PEI on AgNPs and the negative charges of HSA. Loading increases as a function of hydroxystearate content reaching values up to 3.3 wt %, without modifying nanocrystals’ structural parameters, crystallinity, and morphology, which are influenced just by HSA content. Moreover, the good interaction with hydroxystearate functionalized hydroxyapatite crystals stabilizes AgNPs, which show a sustained release in solution.

## Figures and Tables

**Table 1:** Structural parameters, silver content and surface parameters of composite materials. Standard deviations are reported in parentheses.

Sample	a (Å)	c (Å)	τ _002_ (Å)	τ _310_ (Å)	Ag Content (wt %)	ζ Potential (mV)	Contact Angle (°)
HA	9.423(2)	6.883(2)	523(7)	315(4)	--	−8.5	10 (4)
HA-Ag5	9.427(2)	6.883(2)	518(5)	308(3)	0.5 (1)	+0.5	20 (5)
HA-Ag20	9.421(1)	6.879(1)	522(6)	316(4)	1.0 (1)	+7.2	18 (2)
HA-Ag50	9.427(2)	6.886(3)	515(6)	304(3)	1.2 (1)	+8.3	25 (2)
HSA10-Ag5	9.428(3)	6.878(3)	473(5)	212(3)	0.6 (1)	−2.6	113 (2)
HSA10-Ag20	9.430 (2)	6.884 (2)	478(5)	208(2)	2.2 (1)	+3.5	119 (1)
HSA10-Ag50	9.414(2)	6.871(2)	471(4)	200(3)	2.9 (1)	+5.0	114 (3)
HSA20-Ag5	9.431(3)	6.879(3)	450(5)	172(2)	0.8 (1)	−5.0	123 (5)
HSA20-Ag20	9.414(2)	6.871(3)	458(4)	176(2)	2.4 (1)	+1.2	128 (3)
HSA20-Ag50	9.430 (2)	6.880 (2)	453(4)	171(1)	3.3 (1)	+2.0	121 (2)
